# LIGHT/IFN‐γ triggers β cells apoptosis *via* NF‐κB/Bcl2‐dependent mitochondrial pathway

**DOI:** 10.1111/jcmm.12876

**Published:** 2016-05-31

**Authors:** Quan‐You Zheng, Zhao‐Hui Cao, Xiao‐Bo Hu, Gui‐Qing Li, Shi‐Fang Dong, Gui‐Lian Xu, Ke‐Qin Zhang

**Affiliations:** ^1^ Department of NephrologySouthwest HospitalThird Military Medical UniversityChongqingChina; ^2^ Hunan Province Cooperative Innovation Center for Molecular Target New Drug StudySchool of Pharmacy and BiosciencesUniversity of South ChinaHengyangChina; ^3^ Department of ImmunologySchool of Basic Medical SciencesThird Military Medical UniversityChongqingChina

**Keywords:** LIGHT, NF‐κB, mitochondrial stress, apoptosis, pancreatic beta cell

## Abstract

LIGHT recruits and activates naive T cells in the islets at the onset of diabetes. IFN‐γ secreted by activated T lymphocytes is involved in beta cell apoptosis. However, whether LIGHT sensitizes IFNγ‐induced beta cells destruction remains unclear. In this study, we used the murine beta cell line MIN6 and primary islet cells as models for investigating the underlying cellular mechanisms involved in LIGHT/IFNγ – induced pancreatic beta cell destruction. LIGHT and IFN‐γ synergistically reduced MIN6 and primary islet cells viability; decreased cell viability was due to apoptosis, as demonstrated by a significant increase in Annexin V^+^ cell percentage, detected by flow cytometry. In addition to marked increases in cytochrome *c* release and NF‐κB activation, the combination of LIGHT and IFN‐γ caused an obvious decrease in expression of the anti‐apoptotic proteins Bcl‐2 and Bcl‐xL, but an increase in expression of the pro‐apoptotic proteins Bak and Bax in MIN6 cells. Accordingly, LIGHT deficiency led to a decrease in NF‐κB activation and Bak expression, and peri‐insulitis in non‐obese diabetes mice. Inhibition of NF‐κB activation with the specific NF‐κB inhibitor, PDTC (pyrrolidine dithiocarbamate), reversed Bcl‐xL down‐regulation and Bax up‐regulation, and led to a significant increase in LIGHT‐ and IFN‐γ‐treated cell viability. Moreover, cleaved caspase‐9, ‐3, and PARP (poly (ADP‐ribose) polymerase) were observed after LIGHT and IFN‐γ treatment. Pretreatment with caspase inhibitors remarkably attenuated LIGHT‐ and IFNγ‐induced cell apoptosis. Taken together, our results indicate that LIGHT signalling pathway combined with IFN‐γ induces beta cells apoptosis *via* an NF‐κB/Bcl2‐dependent mitochondrial pathway.

## Introduction

Type 1 diabetes mellitus (T1DM) is an organ‐specific autoimmune disorder characterized by chronic inflammation and pancreatic insulin‐producing beta cell destruction. Pancreatic beta cell death is primarily caused by apoptosis 1, 2, 3 resulting due to a number of factors. One of the main factors leading to beta cell apoptosis is the secretion of the pro‐inflammatory cytokine, interferon (IFN)‐γ, by autoreactive T lymphocytes and macrophages invading the islets. Moreover, beta cell apoptosis is driven by specific combinations of cytokines, such as the combination of IFN‐γ and tumour necrosis factor (TNF)‐α, but not by a single cytokine alone 4, 5. The combination and distribution of cytokines vary in different animal models 6, 7. Further understanding of the apoptotic events activated by the combinations of different cytokines in beta cells is necessary for developing individualized therapy strategies to prevent islet beta cell destruction in T1DM.

LIGHT [lymphotoxin (LT)‐like, exhibits inducible expression and competes with HSV glycoprotein D for herpesvirus entry mediator (HVEM), a receptor expressed by T lymphocytes], also known as TNFSF14 (tumour necrosis factor superfamily member 14), is a new member of the TNF superfamily and plays an important role during innate or adaptive immune processes *via* binding to its receptors, lymphotoxin β receptor (LTβR) or HVEM 8, 9, 10, 11. The LIGHT‐LTβR pathway recruits and activates naive T cells in the islets at the onset of diabetes. Early treatment with LTβR‐Ig in non‐obese diabetic (NOD) mice prevents insulitis and insulin‐dependent diabetes mellitus, and LTβR‐Ig treatment at a late stage of insulitis also dramatically reverses insulitis and prevents diabetes 12, 13, 14. Our previous results showed that LIGHT signalling promotes pro‐inflammatory cytokine IFN‐γ production 15. In certain tumour cells, LIGHT binding to LTβR activates the IFNγ‐induced pro‐apoptotic pathway 16, 17, 18, 19. However, it is unclear whether LIGHT sensitizes IFNγ‐induced beta cells apoptosis and what are the possible signal transduction events of LIGHT and IFN‐γ combinations in beta cell apoptosis.

To further understand the activation of apoptotic pathways by the combination of LIGHT and IFN‐γ in beta cells, we used MIN6 insulinoma beta cells and primary islet cells as models. Here, for the first time, these results demonstrate that the LIGHT signalling pathway combined with IFN‐γ triggers beta cell apoptosis *via* an NF‐κB/Bcl2‐dependent mitochondrial pathway.

## Materials and methods

### Cell lines and primary islet cells

MIN6 cells are SV40 T‐transformed insulinoma beta cells. Primary islet cells were isolated from 5 to 8‐week age female NOD mice. The stable MIN6 cells were maintained in 5% CO_2_ at 37°C. Cells were grown in DMEM culture medium containing 25 mM glucose (Gibco, USA), supplemented with 15% FBS (Hyclone, Grand Island, NY, USA), 100 U/ml penicillin, 100 μg/ml streptomycin, and 2 mM glutamine. Cells were treated with 100 ng/ml recombinant mouse IFN‐γ (Peprotech, Rocky Hill, NJ, USA) and various concentrations of recombinant mouse LIGHT (Peprotech). The optimal cytokine concentration of LIGHT for cytotoxic action was 5 μg/ml.

### Assessment of cytokine‐mediated cytotoxicity by MTT assays

Cells were seeded at an initial density of 30,000/well the day before the experiment, and treated with 100 ng/ml IFN‐γ and various concentrations of LIGHT; or 100 ng/ml IFN‐γ or 5 μg/ml LIGHT alone or in combination for 48 h; or 100 ng/ml IFN‐γ, 10 ng/ml TNF‐α, 5 μg/ml LIGHT, or 17.5 ng/ml IL‐1β alone, or IL‐1β in combination with IFN‐γ, TNF‐α or LIGHT for 48 h. In some experiments, MIN6 cells were pretreated with the NF‐κB inhibitor PDTC, or a broad range caspase inhibitor Z‐VAD‐FMK (benzyloxycarbonyl‐Val‐Ala‐Asp fluoromethylketone) (Beyotime Institute of Biotechnology), for 1 h before IFN‐γ and LIGHT combination treatment for 48 h. MTT assays were performed as described previously 5.

### Analysis of cell apoptosis by flow cytometry

To observe morphological changes of live cells under a phase contrast microscope (Olympus 1X71S8F‐2, Tokyo, Japan), MIN6 cells were seeded in 96‐well microtiter plates and treated with IFN‐γ (100 ng/ml) plus LIGHT (5 μg/ml) for 0, 24, and 48 h. To determine cell apoptosis by flow cytometry, cells were treated with media, IFN‐γ (100 ng/ml), or LIGHT (5 μg/ml) alone, or in combination for 24 and 48 h. In some experiments, cells were pretreated with caspase inhibitors Z‐VAD‐FMK for 1 h before LIGHT and IFN‐γ treatment. To determine the expression of HVEM and LTβR on MIN6 cells, cells were incubed with antibodies against HVEM (Biolegend) and LTβR (Biolegend, San Diego, CA, USA), respectively, and analysed by flow cytometry (BD, FACS Canto II). For receptor blockage experiments, cells were pretreated with the recombinant plasmids transfection supernatants containing soluble fusion proteins HVEM‐IgGFc, LTβR‐IgGFc or N66F‐IgGFc, for 1 h before LIGHT and IFN‐γ treatment. FACS was performed as described previously 5.

### Western blot

MIN6 cells were seeded in six‐well plates and treated with the combination of IFN‐γ (100 ng/ml) and LIGHT (5 μg/ml) for 0, 0.5, 1, 12, 24 h or for the indicated times. In some experiments, cells were pre‐incubated with or without the NF‐κB inhibitor PDTC for 1 h and then treated with or without IFN‐γ (100 ng/ml) plus LIGHT (5 μg/ml) for 12 h. Antibodies against cytochrome *c* (BD Pharmingen, Franklin Lakes, NJ, USA), COX4 (BD Pharmingen), NF‐κB p65 (Beyotime Institute of Biotechnology), Bcl‐2 (Cell Signalling, Danvers, MA, USA), Bcl‐xL (Beyotime Institute of Biotechnology, Beijing, China), Bax (Beyotime Institute of Biotechnology), Bak (Beyotime Institute of Biotechnology), caspase‐9 (Beyotime Institute of Biotechnology), PARP (Beyotime Institute of Biotechnology), caspase‐3 (Cell Signalling, Danvers, MA, USA) and cleaved caspase‐3 (Cell Signalling, Danvers) were used to analyse the expression of proteins by Western blot as previously described 5.

### Immunohistochemistry

For immunohistochemistry staining, pancreatic tissues of LIGHT^+/+^ NOD mice and LIGHT^−/−^ NOD mice were fixed in 4% (w/v) paraformaldehyde, routinely processed and embedded in paraffin. The 3–5 μm sections were stained with a monoclonal rabbit anti‐mouse NF‐κB p65 antibody, a polyclonal rabbit anti‐mouse BAK (Beyotime Institute of Biotechnology), and a monoclonal rabbit anti‐mouse BCL‐2 (Cell Signalling) respectively.

### Analysis of NF‐κB activation by the immunofluorescence staining

For immunofluorescence staining of NF‐κB in MIN6 cells, cells were grown on coverslips and treated with or without IFN‐γ (100 ng/ml) plus LIGHT (5 μg/ml) for 1 h. For immunofluorescence staining of NF‐κB in pancreatic tissues of NOD mice, pancreatic tissues were fixed in 4% (w/v) paraformaldehyde, routinely processed and embedded in paraffin. Antibodies against NF‐κB p65 (Beyotime Institute of Biotechnology) were used to analyse the activation of NF‐κB by immunofluorescence staining.

### Measurement of nitrite

MIN6 cells were seeded in 96‐well microtiter plates and treated with media, IFN‐γ (100 ng/ml) or LIGHT (5 μg/ml) alone or in combination for 48 h. Nitric oxide (NO) level in culture supernatant was determined by measuring the levels of nitrite, a stable by‐product of NO by using Griess assay (Beyotime Institute of Biotechnology) according to the manufacturer's suggested protocols.

### Intracellular ROS detection

MIN6 cells were seeded at an initial density of 100,000/well 48‐well tissue culture plates the day before the experiment, and treated with 100 ng/ml IFN‐γ or 5 μg/ml LIGHT alone or in combination for 6 h. The intracellular (reactive oxygen species, ROS) were analysed using a Reactive Oxygen Species Assay Kit (Beyotime Institute of Biotechnology) according to the manufacturer's suggested protocols. The fluorescence intensity was measured by flow cytometry (BD, FACS Canto II).

### Statistics

graphpad prism 5.0 (GraphPad Software, Inc., La Jolla, CA, USA) was used for statistical analysis. Data are presented as mean ± SEM, and statistical analysis of the data was performed by unpaired *t*‐test. Statistically significant differences were assessed at *P* < 0.05.

## Results

### LIGHT/IFN‐γ synergism inhibits pancreatic beta cell viability

**Figure 1:**
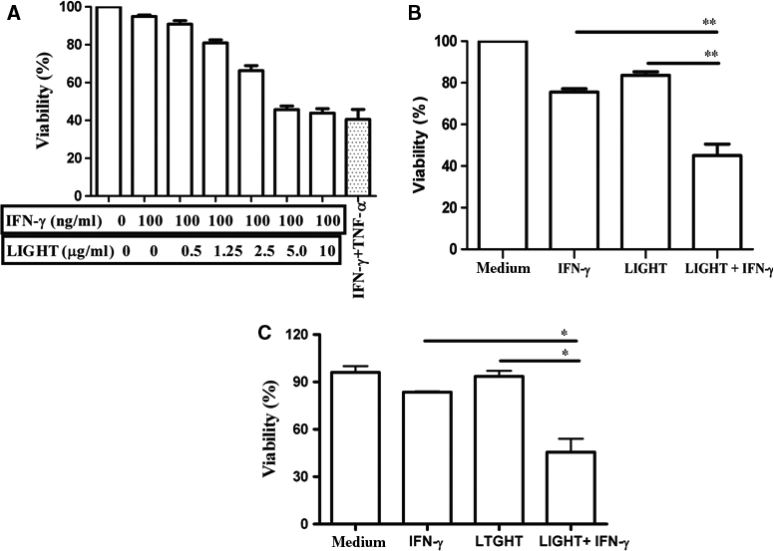
LIGHT and IFN‐γ synergistically inhibit beta cell viability. (**A**) MIN6 cells (3 × 10^4^/well) were seeded in 96‐well microtiter plates and treated with different concentrations of LIGHT in the presence of 100 ng/ml IFN‐γ for 48 h. Treatment with 100 ng/ml IFN‐γ plus 10 ng/ml TNF‐α was regarded as positive control. (**B**) MIN6 cells, or (**C**) islet cells of NOD mice with (3 × 10^4^/well) were seeded in 96‐well microtiter plates and treated with IFN‐γ (100 ng/ml) or LIGHT (5 μg/ml) alone or in combination for 48 h. Cell viability of the aforementioned groups was measured by MTT assay. The OD value was detected at 490 nm. The OD value at 490 nm of the untreated cells was set to 100%. Results are expressed as means ± SEM. *P < 0.05 and **P < 0.01. All experiments were repeated at least three times.

To explore the effect of LIGHT and IFN‐γ combination on pancreatic beta cell destruction, cells were exposed to increasing concentrations of LIGHT in the presence of IFN‐γ for 48 h. Cell viability decreased in a dose‐dependent manner with a peak concentration at 5 μg/ml (Fig. 1A). Although both LIGHT and IFN‐γ alone exhibited some inhibition of cell viability, the combination of LIGHT and IFN‐γ significantly reduced cell viability (Fig. 1B, IFN‐γ *versus* LIGHT+IFN‐γ: *P* = 0.0061; LIGHT *versus* LIGHT+IFN‐γ: *P* = 0.0026) as well as the combination of LIGHT and IL‐1β (Fig. S1). Moreover, both NO formation and ROS generation was obviously augmented in the presence of LIGHT + IFN‐γ (Fig. S2A and B), which might contribute to LIGHT/IFN‐γ‐mediated cell death. A similar effect was observed on primary islet cells of NOD mice (Fig. 1C). Together, these results indicate that LIGHT and IFN‐γ combination treatment synergistically inhibits pancreatic beta cell viability.

### LIGHT and IFN‐γ treatment triggers MIN6 cells apoptosis

**Figure 2:**
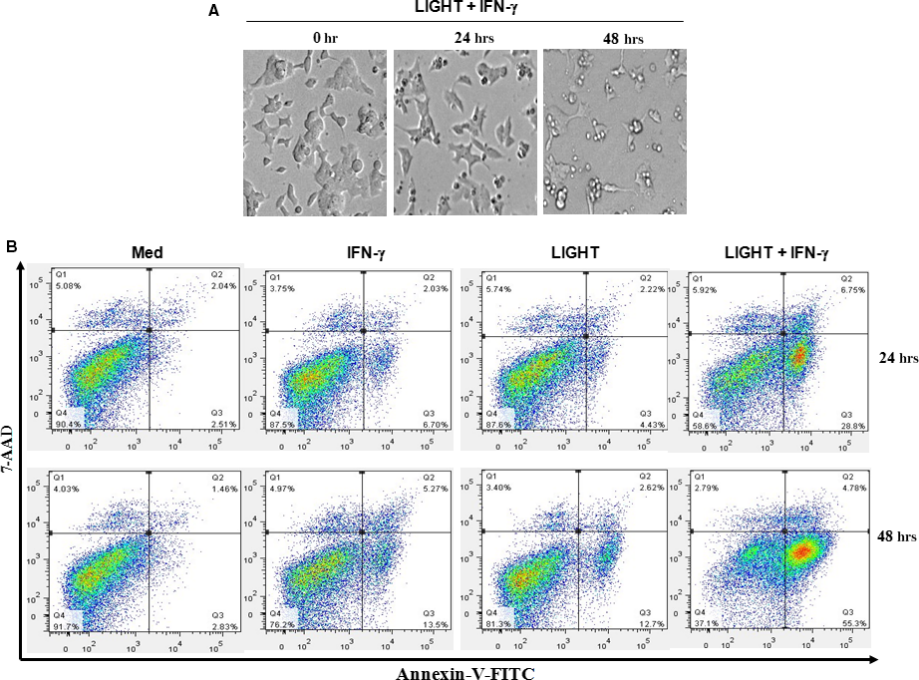
The combination of LIGHT and IFN‐γ treatment induces MIN6 cell apoptosis. (**A**) Cells were treated with IFN‐γ (100 ng/ml) and LIGHT (5 μg/ml) in combination for 0, 24 and 48 h, and were photographed under a phase contrast microscope. Magnification, 100×. (**B**) Cells were treated with media, IFN‐γ (100 ng/ml) or LIGHT (5 μg/ml) alone, or in combination for 24 and 48 h. Cells were double stained with Annexin V‐FITC and 7‐AAD and the percentage of apoptotic cells (Annexin V^+^ and 7‐AAD ^−^ cells) was determined by flow cytometry. Data shown are representative of two‐independent experiments.

Cell morphology was further assessed under a phase contrast microscope after treatment with the combination of LIGHT and IFN‐γ for indicated times. Cell shape changed gradually from sprawling to round, and even floating after treatment for 48 h (Fig. 2A); this change in cell morphology indicated that LIGHT/IFN‐γ synergism‐mediated cell growth inhibition was because of cell death.

Cell death occurs through a number of mechanisms including apoptosis, necrosis and autophagic cell death. To further investigate which type of cell death was induced by LIGHT and IFN‐γ treatment, cells were assayed by flow cytometry through a double labelling assay (Annexin V combined with 7‐AAD), which allowed a clear distinction of necrotic or late apoptotic (Annexin V^+^/7‐AAD^+^) and early apoptotic cells (Annexin V^+^/7‐AAD^−^). After treatment with the combination of LIGHT and IFN‐γ, the early apoptotic cell percentage increased to 28.8% at 24 h and 55.3% at 48 h, but the percentage of late apoptotic or necrotic cells remained below 7.0% (Fig. 2B), demonstrating that LIGHT and IFN‐γ induced cell apoptosis rather than necrosis.

### Combination treatment with LIGHT and IFN‐γ induces mitochondrial stress in MIN6 cells

**Figure 3:**
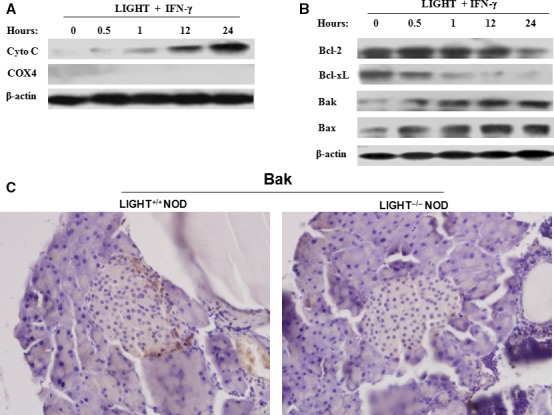
Increased release of cytochrome *c* and alterations in Bcl‐2 family members expression in LIGHT and IFN‐γ‐induced apoptosis of MIN6 cells. Cells were treated with LIGHT (5 μg/ml) and IFN‐γ (100 ng/ml) in combination for the indicated times. Cells were harvested and cytoplasmic protein was extracted. (**A**) Cytochrome *c* release was evaluated by Western blot. Cytochrome oxidase subunit IV (COX4), located exclusively in the mitochondria, was used here to confirm whether the cytoplasmic protein fractions included mitochondrial proteins. (**B**) The expression of Bcl‐2, Bcl‐xL, Bak and Bax was measured by Western blot. Equal protein loading in each lane was confirmed by probing the blots with anti‐β‐actin antibody. Data are representative of two‐independent experiments. (**C**) The expression of Bak in primary islets of 20‐week‐old female LIGHT ^+/+^ and LIGHT ^−/−^ NOD mice was measured by immunohistochemisty. Magnification, 200×.

The classical apoptotic pathways include intrinsic mitochondrial pathways, extrinsic death receptor pathways, and endoplasmic reticulum stress pathways. When combined with TNF‐α, IFN‐γ secreted by activated T lymphocytes is involved in beta cell apoptosis *via* the mitochondrial pathway 20. Moreover, it has been suggested that in certain tumour cells, LIGHT activates an IFN‐γ‐induced pro‐apoptotic pathway through mitochondrial pathways 16, 17, 18, 19, 21. To investigate whether combination treatment with LIGHT and IFN‐γ induces mitochondrial stress in beta cells, we determined the effects of LIGHT and IFN‐γ treatment on mitochondrial cytochrome *c* release at the indicated times. Cytosol cytochrome *c* increased markedly over time after combination treatment of cells with LIGHT and IFN‐γ (Fig. 3A), indicating that cytochrome c was released from mitochondrion to cytosol after stimulation with LIGHT and IFN‐γ.

### Combination treatment with LIGHT and IFN‐γ leads to altered expression of Bcl‐2 family members

The release of cytochrome *c* from mitochondria is regulated by the Bcl‐2 family of proteins 22. Transcriptional and post‐transcriptional modification and protein–protein interactions between members of the Bcl‐2 family determine the fate of cells in this pathway 23, 24. To explore the effect of LIGHT and IFN‐γ treatment on Bcl‐2 family members, we assessed the levels of these proteins. After combination treatment with LIGHT and IFN‐γ, expression of the anti‐apoptotic molecules Bcl‐2 and Bcl‐xL was obviously down‐regulated, while that of the pro‐apoptotic molecules Bak and Bax markedly increased over time (Fig. 3B). Consistent with the *in vitro* results, compared with that of LIGHT^+/+^ NOD mice, a decrease in Bak expression and peri‐insulitis was observed in islets of LIGHT^−/−^ NOD mice (Fig. 3C). These results suggest that LIGHT and IFN‐γ treatment enhances cytochrome *c* release, which may be attributed to a decrease in the ratio of anti‐apoptotic molecules, such as Bcl‐xL, to pro‐apoptotic molecules, such as Bax, subsequently changing mitochondrial membrane structure.

### LIGHT and IFN‐γ treatment is involved in NF‐κB activation in beta cells

**Figure 4:**
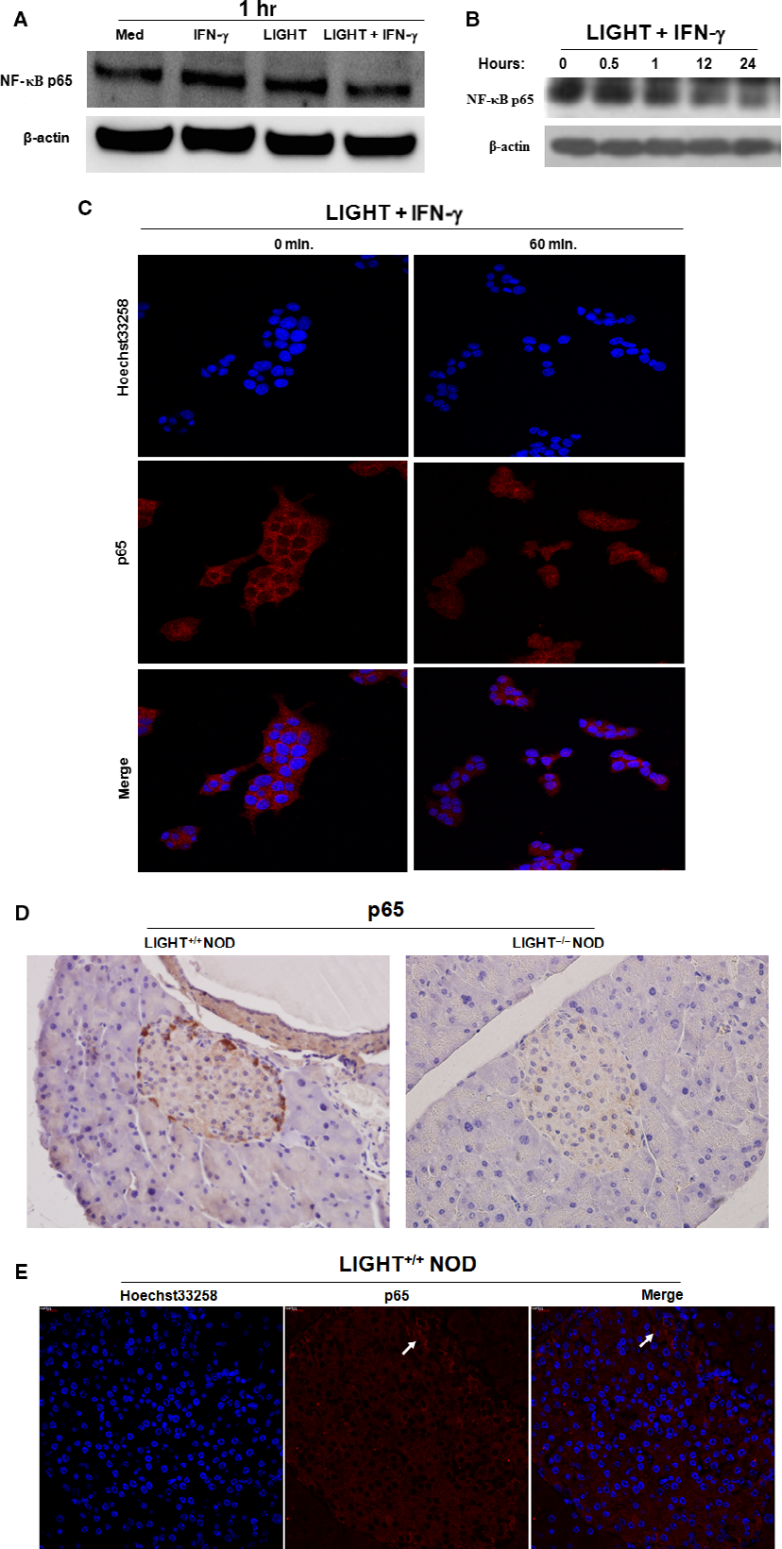
NF‐κB activation is involved in beta cell destruction. (**A**) and (**B**) MIN6 cells were treated with LIGHT (5 μg/ml) or IFN‐γ (100 ng/ml) alone, or in combination for the indicated times. Cytoplasmic NF‐κB p65 levels were determined by Western blot. Equal protein loading in each lane was confirmed by probing the blots with anti‐β‐actin antibody. (**C**) MIN6 cells were treated with LIGHT (5 μg/ml) and IFN‐γ (100 ng/ml) in combination for indicated times. The nuclear localization of NF‐κB p65 was determined by confocal laser scanning microscopy. Nuclei were stained with Hoechst 33258 solution (5 μg/ml). (**D**) The expression of NF‐κB p65 in primary islets of 20‐week‐old female LIGHT ^+/+^ and LIGHT ^−/−^ NOD mice was measured by immunohistochemistry. Magnification, 200×. (**E**) The nuclear localization of NF‐κB p65 in LIGHT ^+/+^ NOD mice with (peri)insulitis was further determined by confocal laser scanning microscopy. Nuclei were stained with Hoechst 33258 solution (5 μg/ml).

NF‐κB is actively involved in beta cell death 25 and LIGHT‐LTβR signalling can activate NF‐κB signalling pathways in some cell types 21. To further investigate the upstream events occurring during mitochondrial stress induced by the combination of LIGHT and IFN‐γ treatment, we considered the potential involvement of NF‐κB. Cytoplasmic NF‐κB p65 protein levels 1 h after cell exposure to LIGHT and IFN‐γ were comparable to those of cells in medium (Fig. 4A), but then decreased in a time‐dependent manner (Fig. 4B); levels of NF‐κB p65 in the nuclei increased markedly (Fig. 4C), suggesting that NF‐κB was activated and translocated from cytosol to nucleus. Accordingly, NF‐κB p65 expression and activation decreased in LIGHT^−/−^ NOD mice (Fig. 4D and E). These results demonstrate that NF‐κB activation is associated with LIGHT and IFN‐γ treatment.

### Inhibition of NF‐κB activation reduces LIGHT/IFNγ‐mediated beta cells apoptosis

**Figure 5:**
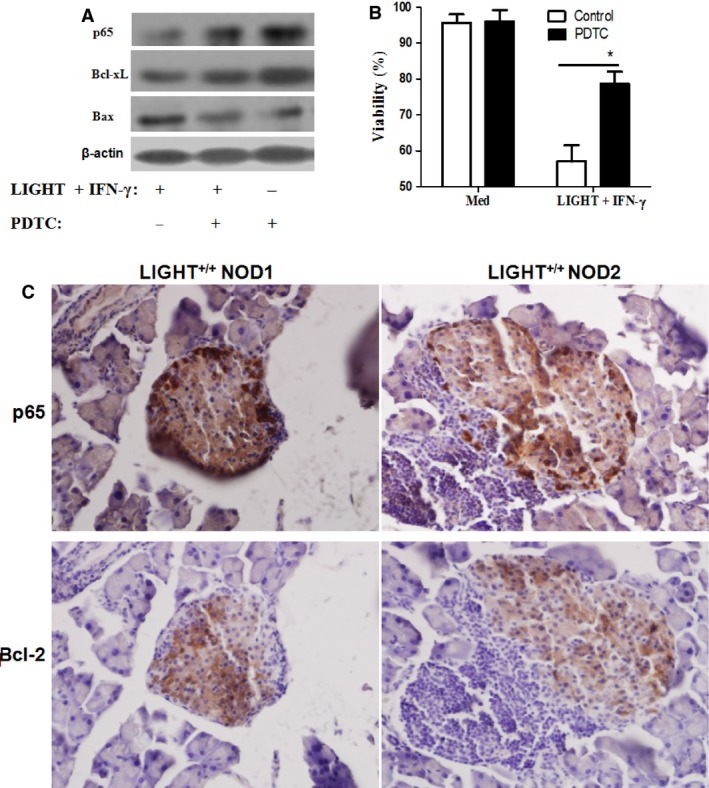
Beta cell destruction is NF‐κB/Bcl‐2 pathway dependent. (**A**) After pretreatment with or without PDTC (50 μM), an NF‐κB inhibitor, for 1 h, MIN6 cells were treated with a combination of IFN‐γ and TNF‐α for 12 h. The expression of cytoplasmic NF‐κB p65, Bcl‐xL, and Bax was determined by Western blot. Equal protein loading in all lanes was confirmed by probing the blots with anti‐β‐actin antibody. (**B**) After pretreatment with or without PDTC (50 μM) for 1 h, MIN6 cells were treated with or without the LIGHT and IFN‐γ combination for 48 h. Cell viability was measured by MTT assay. (**C**) Reverse expression of p65 and Bcl‐2 was observed in primary islets in NOD mice with (peri) insulitis by immunohistochemisty. [Left and right represent different fields in 2 NOD mice with (peri) insulitis]. Magnification, 200×. Data are expressed as mean ± SEM. *P < 0.05. Data are representative of two‐independent experiments.

To further assess the relationship between dysregulated expression of anti‐apoptotic Bcl‐xL and pro‐apoptotic Bax and NF‐κB activation, cells were pretreated with PDTC (an effective NF‐κB inhibitor) for 1 h and then treated with the combination of LIGHT and IFN‐γ for 12 h. PDTC treatment reversed cytokine‐induced Bcl‐xL down‐regulation and Bax up‐regulation (Fig. 5A), and markedly increased cell viability (Fig. 5B). Moreover, it was interesting that we observed a completely contrast expression pattern of NF‐κB with that of Bcl‐2, which shows a higher expression of NF‐κB while a lower Bcl‐2 expression in the periphery of prediabetic NOD mice with insulitis (Fig. 5C). These data suggest that the NF‐κB/Bcl‐2 pathway is involved in LIGHT and IFN‐γ‐induced MIN6 cell apoptosis.

### Combination of LIGHT and IFN‐γ induces caspase‐9 and caspase‐3 activation

**Figure 6:**
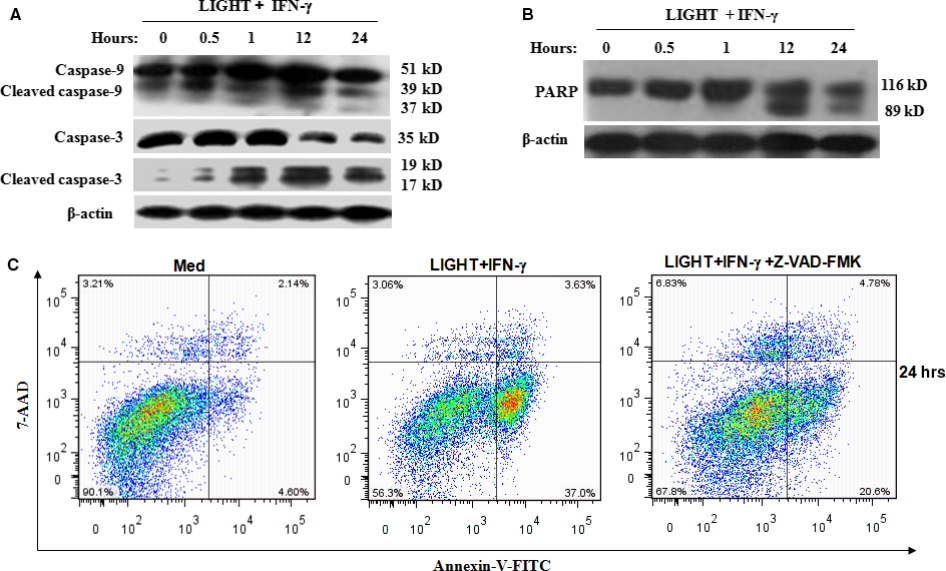
LIGHT and IFNγ‐induced MIN6 cells apoptosis is caspase‐dependent. Cells were treated with IFN‐γ (100 ng/ml) and LIGHT (5 μg/ml) in combination for 0.5, 1, 12 and 24 h. The expression of caspase‐3, ‐9, cleaved caspase‐3, ‐9 (**A**), and cleaved PARP (**B**) was measured by Western blot. Equal protein loading in all lanes was assessed by probing the blots with β‐actin antibody. (**C**) After pretreatment with or without Z‐VAD‐FMK (50 μM), a broad range caspase inhibitor, for 1 h, cells were treated with IFN‐γ and LIGHT in combination for 48 h. Cells were double stained with annexin V and 7‐AAD and then analysed by flow cytometry. Data are representative of three independent experiments.

Studies have shown that caspase‐9 plays crucial roles in the mitochondrial apoptotic pathway, and it is activated by cytochrome *c* 22, 26. Cleavage of caspase‐3 is one of the downstream events following caspase‐9 activation. To investigate if caspase‐9 and caspase‐3 activation is involved in LIGHT‐ and IFN‐γ‐mediated MIN6 cell apoptosis, the expression of cleaved caspase‐9 and ‐3 was analysed by Western blot. As illustrated in Fig. 6A, the combination of LIGHT and IFN‐γ caused time‐dependent cleavage of caspase‐3 and caspase‐9. Poly (ADP‐ribose) polymerase (PARP), a nuclear poly‐polymerase, is one of the main cleavage targets of caspase‐3, and served as a marker of cell apoptosis 27. As shown in Fig. 6B, the cleaved PARP fragment was observed beginning 12 h after cell exposure to the combination of LIGHT and IFN‐γ, which may be also associated with high NO formation induced by the LIGHT and IFN‐γ treatment (Fig. S2A).

To further verify that caspase protein activation is necessary for LIGHT and IFN‐γ‐ induced MIN6 cell apoptosis, cells were pretreated with Z‐VAD‐FMK (a broad range caspase inhibitor) before cytokine treatment. As shown in Fig. 6C, LIGHT and IFN‐γ‐induced cell apoptosis was obviously reduced by Z‐VAD‐FMK pretreatment (37% *versus* 20.6% at 24 h), suggesting that caspase activation is required to trigger apoptosis in MIN6 cells.

### Blockage of LIGHT activity with its soluble receptor fusion proteins attenuates LIGHT/IFN‐γ synergism‐mediated cell apoptosis

**Figure 7:**
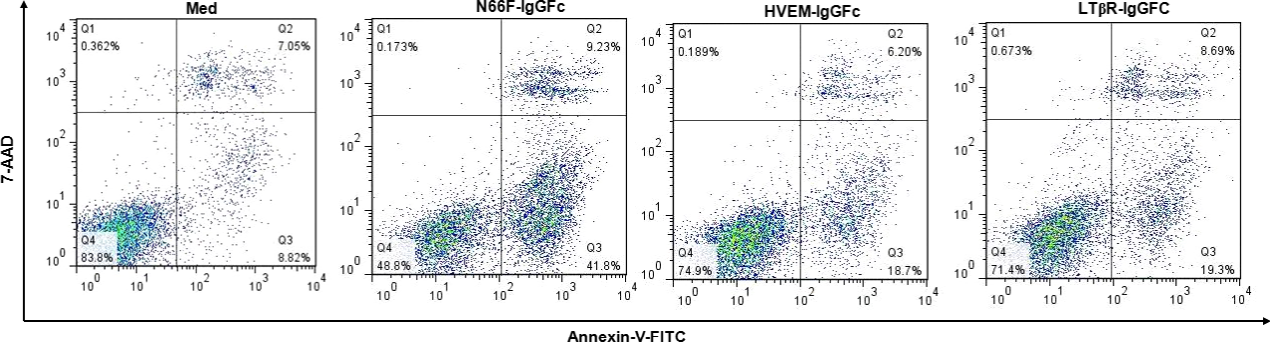
Blockage of LIGHT activity by soluble receptor fusion proteins reverses LIGHT/IFN‐γ synergism‐mediated MIN6 cell apoptosis. After preincubation with soluble receptor fusion proteins HVEM‐Ig or LTβR‐Ig or N66F‐Ig (a negative control, which is an Fc fusion protein that does not interfere with LTβR/HVEM/LIGHT interactions), cells were treated with IFN‐γ and LIGHT in combination for 48 h. Cells were double stained with annexin V and 7‐AAD and then analysed by flow cytometry. Data are representative of two‐independent experiments.

Considering that MIN6 cells express both LTβR and HVEM (Fig. S3), we further distinguished the cytotoxic functions of LIGHT in beta cell apoptosis specifically through HVEM and/or LTβR by pretreating cells with soluble receptor fusion proteins HVEM‐Ig or LTβR‐Ig before cytokine treatment. Compared with the treatment with N66F‐Ig (a negative control, which is a Fc fusion protein that does not interfere with LTβR/HVEM/LIGHT interactions), HVEM‐Ig or LTβR‐Ig treatment caused a remarkable decrease in LIGHT and IFN‐γ‐mediated MIN6 cell apoptosis (Fig. 7, N66F‐IgGFc *versus* HVEM‐IgGFc: 41.8% *versus* 18.7%, respectively; N66F‐IgGFc *versus* LTβR‐IgGFc: 41.8% *versus* 19.3%, respectively), suggesting that LIGHT plays a role in IFN‐γ‐mediated MIN6 cell apoptosis, at least partially, *via* both HVEM and LTβR signalling pathways.

## Discussion

Cytokines released by infiltrative T lymphocytes and macrophages are considered the main factors leading to beta cell apoptosis. Moreover, beta cell apoptosis is induced by various combinations of cytokines, with IFN‐γ and TNF‐α being the most likely cytokines to act synergistically during the inflammation of pancreatic beta cells 28.

Like TNF‐α, LIGHT also belongs to the TNF superfamily. LIGHT can enhance IFN‐γ secretion by promoting T‐cell proliferation and activation, and further increase the cytotoxic activity of T lymphocytes in several autoimmune diseases 29, 30. LIGHT functions to induce cancer cell apoptosis, especially in the presence of IFN‐γ 16, 17, 18, 19. However, the effect and underlying mechanism of the combination of LIGHT and IFN‐γ during pancreatic beta cell death are not understood. In this study, we investigated the effect of LIGHT and IFN‐γ on pancreatic beta cell apoptosis by using the murine pancreatic beta MIN6 cell line combined with the primary islets cell in NOD mice as a model.

Similar to the effect of TNF‐α, LIGHT alone exhibited very mild cytotoxicity for MIN6 cells, but when combined with IFN‐γ, LIGHT obviously inhibited cell viability through induction of apoptosis, which is in agreement with the studies in cancer cells 16, 17, 18, 19. In certain tumour cells, LIGHT binding to LTβR activates the IFN‐γ‐induced pro‐apoptotic pathway through mitochondrial pathways 16, 17, 18, 19, 21. Caspase‐3, activated by the initiator caspase‐9, is an executive caspase, which receives the apoptosis signal from the mitochondria 31. Our results found that after cells were treated with a combination of LIGHT and IFN‐γ, cytochrome c release dramatically increased over time, followed by the activation of caspase‐9 and caspase‐3. These results demonstrate that intrinsic mitochondrial pathways are involved in LIGHT and IFN‐γ‐induced MIN6 cell apoptosis.

The mitochondrial apoptotic pathway is tightly controlled by pro‐ and anti‐apoptotic members of the Bcl‐2 family 23, 24. However, the dependence of cytokine‐induced beta cell apoptosis on Bcl‐2 family proteins is still controversial. In one study, TNF‐α and IFN‐γ treatment of MIN6 insulinoma cells did not significantly influence Bcl‐2 28. IL‐1β‐ and IFN‐γ‐induced beta cell death was demonstrated to be Bax‐independent in INS‐1 cells and rat islets 32; Zhang *et al*. found that Bax is unaltered by LIGHT and IFN‐γ treatment in HT‐29 cells 17. However, Grunnet *et al*. observed an effect of Bax activity on cytokine‐induced beta cell apoptosis in INS‐1 cells and human islets 6. Consistent with the results of a study by Grunnet *et al*., in a previous study, we also found that in TNF‐α and IFN‐γ‐induced NIT‐1 cells, Bax expression is remarkably up‐regulated, while Bcl‐2 expression is gradually down‐regulated 4. In this study, our results show altered expression of Bcl‐2 family proteins (including Bcl‐2, Bcl‐xL, Bax, and Bak) in LIGHT and IFN‐γ‐treated MIN6 cells and islets of prediabetic NOD mice with peri‐insulitis. The increased expression of pro‐apoptotic molecules Bax and Bak, and decreased expression of anti‐apoptotic molecules Bcl‐2 and Bcl‐xL may play an intermediate role in LIGHT and IFN‐γ‐mediated mitochondrial dysfunction in MIN6 cells. The differences among these studies may be related to cell types and the varieties of cytokine combinations.

It remains unclear how LIGHT signalling is involved downstream of the mitochondrial pathway, leading to altered expression of Bcl‐2 family members, and ultimately mitochondrial dysfunction. The NF‐κB signalling pathway has been shown to regulate expression of multiple genes that play key roles in cell survival or apoptosis 33, 34. In some cases, NF‐κB activation inhibits cell death, but can promote cell death in certain cell types, such as in beta cells 5, 25 and gastric epithelial cells 35. Our results demonstrate that LIGHT combined with IFN‐γ leads to a decrease in cytoplasmic NF‐κB p65 levels, but an increase in nuclear NF‐κB p65 expression in MIN6 cells, suggesting that NF‐κB is activated by IFN‐γ and LIGHT treatment. Pretreatment of MIN6 cells with the NF‐κB inhibitor PDTC augmented the rate of Bcl‐xL/Bax, and increased cell viability, demonstrating that NF‐κB‐mediated down‐regulation of Bcl‐xL and up‐regulation of Bax are involved in mediating LIGHT and IFN‐γ‐induced MIN6 cell apoptosis.

Notably, consistent with the findings observed in LIGHT‐ and IFNγ‐mediated apoptosis of MDA‐MB‐231 breast cancer cells 16, although extensive caspase activation of caspases‐3, ‐8 (data not shown) and ‐9, were observed, treatment with a broad range caspase inhibitor and a caspase‐3 inhibitor (data not shown) do not completely block LIGHT/IFNγ‐induced apoptosis in MIN6 cells. It was proposed that a caspase‐independent apoptosis pathway might exist in LIGHT‐ and IFNγ‐mediated MIN6 cell apoptosis; based on our data and that of others 36, 37, 38, the existence of such a pathway may warrant further investigation.

## Conclusion

**Figure 8:**
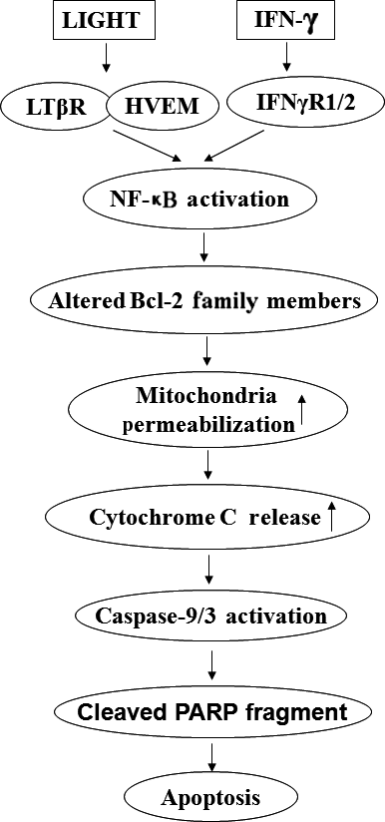
NF‐κB/Mitochondria pathway is involved in beta cells apoptosis induced by LIGHT in the presence of IFN‐γ. LIGHT signalling activates NF‐κB‐mediated mitochondrial pathway *via* the regulation of Bcl‐2 family member's expression, followed with mitochondrial permeabilization, and cytochrome c release, and caspase‐9 activation. Caspase‐9 then activates caspase‐3. The signal from caspase‐3 is transmitted to PARP and then leads to cells apoptosis.

In summary, our results show for the first time that LIGHT combined with IFN‐γ induces islet beta cells apoptosis *via* the intrinsic mitochondrial pathway and that this induction is brought about through NF‐κB‐mediated regulation of anti‐apoptotic or pro‐apoptotic Bcl‐2 family members' expression (Fig. 8). These results broaden our understanding of anti‐ and pro‐apoptotic signalling induced by cytokines and may provide a basis for the development of future intervention strategies for islet graft failure and T1DM.

## Disclosure/conflict of interest

The authors confirm that there are no conflicts of interest.

## Supporting information


**Fig. S1.** IL‐1β in combination with IFN‐γ, TNF‐α or LIGHT synergistically inhibits beta cell viability.

**Fig. S2.** The combination of LIGHT and IFN‐γ treatment augments NO and intracellular ROS production in MIN6 cells.

**Fig. S3.** Expression of LTβR and HVEM on MIN6 cells.
