# Supplementary figures and images for: LIGHT/IFN‐γ triggers β cells apoptosis via NF‐κB/Bcl2‐dependent mitochondrial pathway
Source: J Cell Mol Med. 2016 May 31;20(10):1861–71. doi: 10.1111/jcmm.12876 (PMC5020636; doi:10.1111/jcmm.12876)

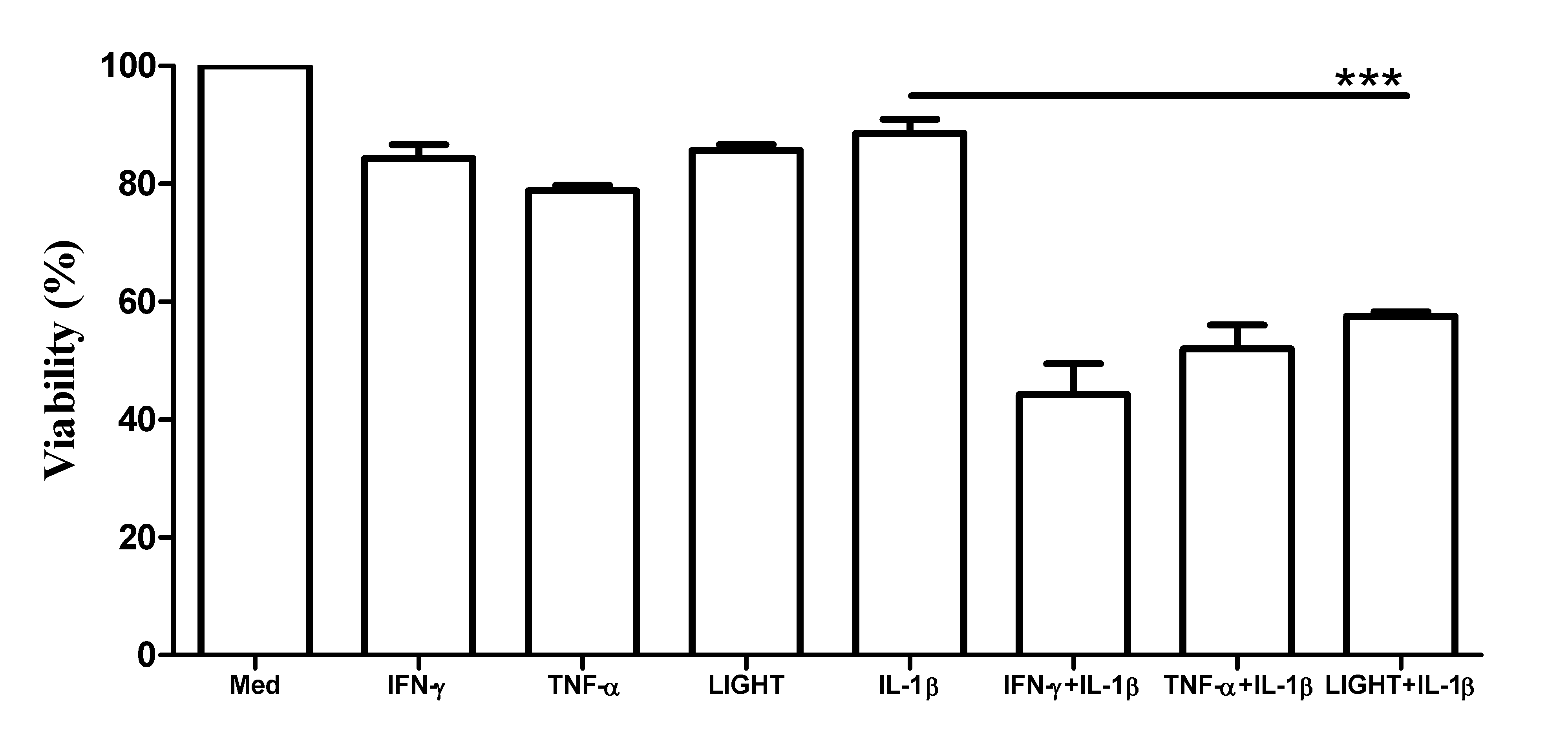

Supplement: Supplementary file 1 — Fig. S1. IL‐1β in combination with IFN‐γ, TNF‐α or LIGHT synergistically inhibits beta cell viability. [file JCMM-20-1861-s001.tif]

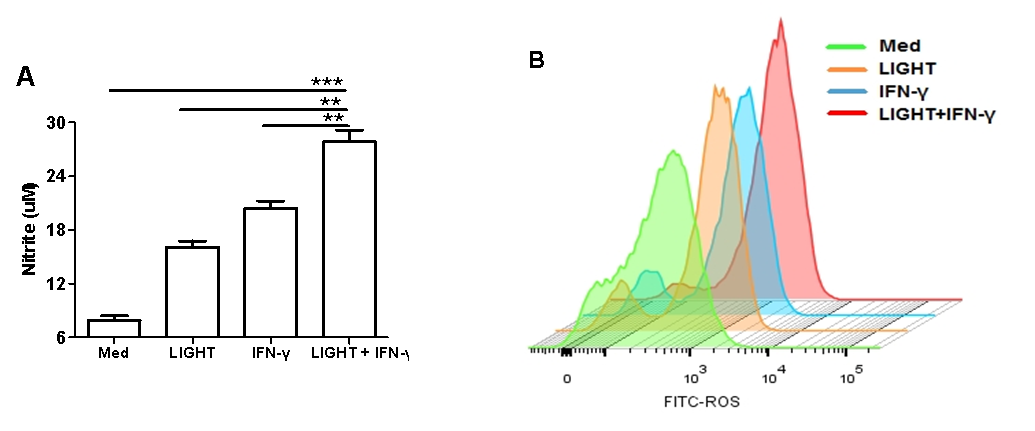

Supplement: Supplementary file 2 — Fig. S2. The combination of LIGHT and IFN‐γ treatment augments NO and intracellular ROS production in MIN6 cells. [file JCMM-20-1861-s002.tif]

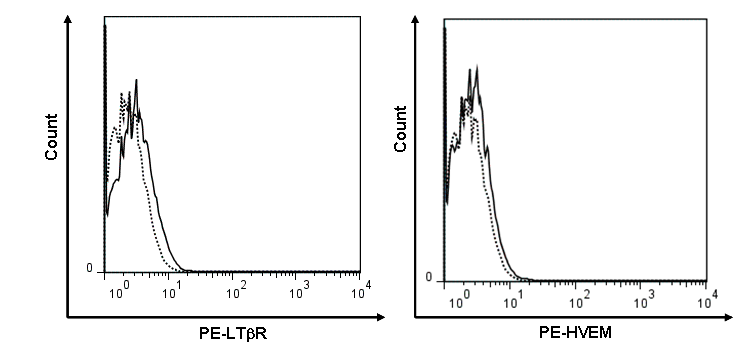

Supplement: Supplementary file 3 — Fig. S3. Expression of LTβR and HVEM on MIN6 cells. [file JCMM-20-1861-s003.tif]
